# From Antarctica to cancer research: a novel human DNA topoisomerase 1B inhibitor from Antarctic sponge *Dendrilla antarctica*

**DOI:** 10.1080/14756366.2022.2078320

**Published:** 2022-05-23

**Authors:** Alessio Ottaviani, Joshua Welsch, Keli Agama, Yves Pommier, Alessandro Desideri, Bill J. Baker, Paola Fiorani

**Affiliations:** a Department of Biology, University of Rome Tor Vergata, Rome, Italy; b Department of Chemistry, University of South Florida, Tampa, FL, USA; c Laboratory of Molecular Pharmacology, Center for Cancer Research, National Cancer Institute, Bethesda, MD, USA; d Institute of Translational Pharmacology, National Research Council, CNR, Rome, Italy

**Keywords:** Natural product, topoisomerase, cancer, drug development

## Abstract

Nature has been always a great source of possible lead compounds to develop new drugs against several diseases. Here we report the identification of a natural compound, membranoid G, derived from the Antarctic sponge *Dendrilla antarctica* displaying an *in vitro* inhibitory activity against human DNA topoisomerase 1B. The experiments indicate that membranoid G, when pre-incubated with the enzyme, strongly and irreversibly inhibits the relaxation of supercoiled DNA. This compound completely inhibits the cleavage step of the enzyme catalytic mechanism by preventing protein binding to the DNA. Membranoid G displays also a cytotoxic effect on tumour cell lines, suggesting its use as a possible lead compound to develop new anticancer drugs.

## Introduction

Despite the advance in clinical research, the fight against cancer still has a long way to go. Nowadays, new technologies rely primarily on finding targets as tumour specific antigens (TSA) that are rare and do not always show an expression level sufficient to make the therapy effective^1^. On the other hand tumour associated antigens (TAA) are overexpressed on cancer cells but also present on normal cells, with the obvious consequence that treatment will affect normal cells as well^2^. For this reason, the identification of drugs with minimal side effects are fundamental. A well characterised tumour target is represented by human DNA topoisomerases, a class of enzymes involved in solving topological DNA problems that occur during fundamental cellular process such as DNA replication, transcription, and chromosome segregation^3–7^. Human DNA topoisomerase IB (htop1), is a monomeric enzyme that relaxes supercoiled DNA cutting a single DNA strand through a concerted mechanism^3^^,^^8–10^. After DNA relaxation occurs, the double-stranded DNA is restored by reformation of the DNA and the enzyme is released^11^. This enzyme is the unique target of campthotecin (CPT) and its derivatives^12^, that intercalate in the nicked DNA preventing the DNA religation step, thus acting as a poison arresting the DNA replicative process and creating double strand breaks that, if not repaired, lead cell to death^13^^,^^14^.

Other compounds, called inhibitors, act by targeting htop1 by preventing either the enzyme from binding to DNA or by preventing the cleavage reaction^15^^,^^16^. Some of them are natural products (NPs)^17^ such as berberine^18^, benzoxazines^19^ and compounds coordinated with metals such as zinc copper and vanadium^20^^,^^21^. In the last few years attention has been focussed on characterising NPs from organisms that live under extreme condition that could permit the development of metabolites with new therapeutic properties^17^^,^^22^.

**Figure 1.**
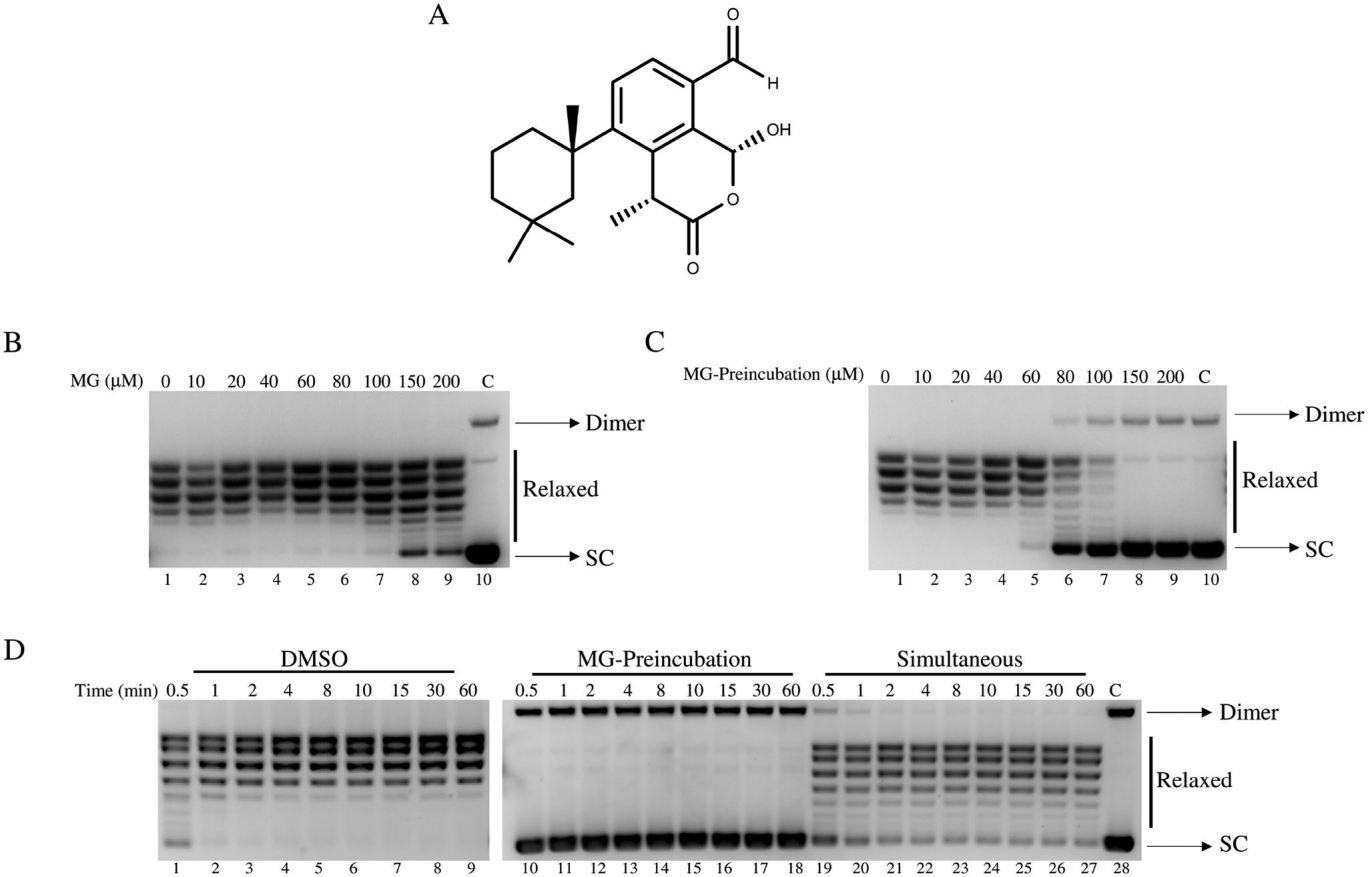
Relaxation of supercoiled DNA. (A) Membranoid G structure. (B) Relaxation of negative supercoiled DNA plasmid by htop1 at increasing MG concentrations (lanes 2–9), lane 1, no drug added and lane 10, no protein added. (C) Pre-treatment of htop1 with MG, at increasing concentrations, before addition of supercoiled DNA plasmid (lanes 2–9), lane 1, no drug added and lane 10, no protein added (D) Relaxation of negative supercoiled DNA plasmid in a time course experiment with DMSO (lanes 1–9), with 100 μM MG in pre-incubation condition (lanes 10–18), and in simultaneous condition (lanes 19–27), lane 28, no protein added. The reaction products are resolved on agarose gel and visualised with ethidium bromide. Dimer indicates dimer supercoiled DNA plasmid; SC indicates super-coiled DNA plasmid.

With this idea in mind we have started screening a library of 60 isolated NPs against htop1, coming from the marine and Antarctic worlds since these types of environment have selected organisms adapted to extreme life conditions, producing NPs with no counterparts in the terrestrial world^22–24^. Of the eight compounds displaying activity at 200 μM (data not show), membranoid G (MG), from Antarctic sponge *Dendrilla antarctica*^25^, (Figure 1(A)), was selected for further characterisation to investigate the mechanism of inhibition due to its unique scaffold, drug-like properties, and abundance of available material. This metabolite is able to inhibit the cleavage reaction of htop1 by binding to the enzyme and preventing the interaction between the htop1 and the DNA substrate. This compound has shown a cytotoxic effect on cancer cell lines having a fast duplication activity, suggesting it as a possible novel antitumor drug targeting htop1.

## Material and methods

### Reagents and drugs

Recombinant htop1 protein (Cat. No. ENZ-306) was purchased from Prospec (Hamada St. 8 Rehovot 7670308 Israel). Topotecan (Hycamtin) was purchased from GlaxoSmithKline (Brentford, Middlesex, TW8 9GS, United Kingdom). MTT (, 3‐(4,5‐Dimethyl‐2‐thiazolyl)‐2,5‐diphenyl‐2H‐tetrazolium bromide, and dimethyl sulfoxide (DMSO) were purchased from MERCK (Darmstadt, Germany). DNA Oligonucleotides CL14-FITC (5′‐GAAAAAAFITCGACTTAG‐3′) labelled with fluorescein isothiocyanate (FITC) at its 5′ end, CP25-P phosphorylated at its 5′ end (5′-TAAAAATTTTTCTAAGTCTTTTTTC‐3′) and R-11 (5′ - GAAAAAATTTT) were purchased from Eurofins Genomics (Sportparkstrasse 2, 85560 Ebersberg, Germany).

### Membranoid G isolation and characterization

In 2020, Shilling et al. outlined the isolation of aplysulfurin and subsequent semisynthetic methanolysis reaction that yielded membranoid G (MG)^25^. Briefly, the freeze-dried sample of the Antarctic sponge *Dendrilla antarctica* was extracted using ACS grade methylene chloride (CH_2_Cl_2_). Reverse phase high performance liquid chromatography (HPLC) yielded aplysulfurin, which was treated with methanol (MeOH) to produce semisynthetic membranoids. Purification of the membranoids was performed using normal phase HPLC resulting in the isolation of 0.7 mg MG from 24 mg of aplysulfurin. Spectroscopic analysis was achieved by nuclear magnetic resonance, mass spectrometry and X-ray diffraction^25^.

### Cells and culture conditions

Dulbecco’s modified Eagle’s medium high glucose, RPMI 1640 medium, foetal bovine serum (FBS), L-glutamine, penicillin/streptomycin, were purchased from Euroclone (Pero, Italy). Complete media (CM) were supplemented with 10% FBS, 2 mM L‐glutamine, 0.1 mg/mL streptomycin, and 100 U/mL penicillin. The ovarian cancer cell line SKOV‐3 was purchased from Cell Biolabs Inc., and maintained in DMEM‐high glucose, CM. Colorectal adenocarcinoma cell line, CACO‐2, colorectal carcinoma cell line HCT-116 and melanoma cell SK-MEL-28, were maintained in RPMI 1640 CM. Non‐small‐cell lung cancer cell line, A‐549, triple negative breast cancer cell SUM-159 and MDA-MB-231, HER2/c-erb-2 positive breast cancer cell line SK-BR-3, and adenocarcinoma HeLa cell line were maintained in DMEM-high glucose, CM. The cells were tested for mycoplasma using the PCR detection Kit (Euroclone). The cells were kept in culture for a maximum of eight passages.

### Htop1 purification

Htop1 for agarose based assays was purified as previously described^16^. Briefly the enzyme gene sequence was cloned in a single copy plasmid and transformed in top1 null EKY3 yeast strain. Transformed cells were grown on SC‐Uracil, with 2% dextrose and then in SC‐Uracil with 2% raffinose until an optical density of A600 = 1. Protein production was induced with 2% galactose for 6 h. Cells were disrupted using glass beads and the enzyme isolated by affinity chromatography. In order to test the integrity of the protein, the fractions were analysed by SDS‐PAGE and immunoblotting.

### Dose dependent and time course relaxation assays

The minimal inhibiting dose of MG on htop1 was assessed through a dose dependent relaxation assay of negatively supercoiled DNA pBlueScript KSII (‐). The reaction was carried out in a final volume of 30 μL containing a buffer composed of 20 mM Tris‐HCl pH 7.5, 0.1 mM EDTA, 10 mM MgCl_2_, 5 μg/mL acetylated bovine serum albumin hereafter indicated as TOPOmix 1X, 150 mM KCl, 1 U of purified htop1, 0.5 μg pBlueScript and different concentrations of MG. As positive control the enzyme was incubated with the same amount of DMSO used to dissolved MG. The reaction was stopped after 1 h incubation at 37 °C by adding 0.5% SDS stop dye. The same procedure was performed for pre-incubation experiment, but before adding the DNA, htop1 was pre-treated for 5 min at 37 °C with different concentrations of MG. For time course experiment the mix previously described was incubated with 100 μM of MG and reactions were stopped at different time points with SDS. In pre-incubation experiments purified htop1 was incubated with 100 μM of MG for 5 min at 37 °C before adding the supercoiled DNA. All samples were resolved on 1% agarose gel and in TBE 1 X buffer containing 48 mM Tris, 45.5 mM boric acid, 1 mM EDTA. The enzyme’s ability to relax supercoiled DNA was visualised through a UV transilluminator after a gel staining in 0.5 μg/mL ethidium bromide and destaining in dH_2_O.

### Cleavage kinetic and htop1-mediated DNA cleavage reactions

In order to analyse the cleavage kinetics CL14-FITC was annealed to a CP25-P complementary oligonucleotide to produce the cleavage substrate, hereafter indicated as suicide substrate (SS). The cleavage reaction was carried out at different time points in presence of 100 μM MG by incubating 0.6 pmol of SS with 1.2 pmol htop1 (Prospec) as elsewhere described with slight modification^26^. After adding the enzyme, aliquots of 30 μl were removed at different times and the reactions were stopped by adding 0.5% SDS (final concentration). In pre-incubation experiment the enzyme was incubated with MG for 5 min at 25 °C prior the addiction of the SS. After a precipitation with ethanol, samples were resuspended in 5 μl of 1 mg/ml of trypsin and incubated at 37 °C for 1 h. The samples were analysed by electrophoresis on denaturing polyacrylamide gel (7 M urea, 20% Acrylamide). For htop1-mediated DNA cleavage reactions a 3′-[32P]-labelled 117-bp DNA oligonucleotide was prepared as previously describe^27^. The oligonucleotide contains previously identified htop1 cleavage sites in 161-bp pBluescript SK(–) phagemid DNA. Approximately 2 nM radiolabelled DNA substrate was incubated with recombinant htop1 in 20 μl of reaction buffer [10 mM Tris-HCl (pH 7.5), 50 mM KCl, 5 mM MgCl_2_, 0.1 mM EDTA, and 15 μg/mL BSA] at 25 °C for 20 min in the presence of various concentrations of membranoid G, with or without 10 µM CPT. The reactions were terminated by adding SDS (0.5% final concentration) followed by the addition of two volumes of loading dye (80% formamide, 10 mM sodium hydroxide, 1 mM sodium EDTA, 0.1% xylene cyanol, and 0.1% bromophenol blue). Aliquots of each reaction mixture were subjected to 20% denaturing PAGE. Gels were dried and visualised using a phosphoimager and ImageQuant software (Molecular Dynamics).

The percentage of cleaved substrate for fluorescent experiment was evaluated by densitometry analysis using ImageLab software. Plots represent the mean of three independent experiments analysed by a two‐way ANOVA test using GraphPad Prism with mean ± SD values. ^****^p < 0.0001 and ***p < 0.001.

### EMSA

DNA mobility shift assay was performed by slightly modifying a previously described procedure^28^. Briefly, 0.1 μg of pBlueScript KSII (‐) supercoiled DNA was incubated in 20 μL reaction with 1 X TOPOmix, 4 U of purified htop1, 15 mM KCl, 1 mM DTT in the absence or in presence of MG at 37 °C for 30 min, or pre-incubating the enzyme with 400 μM MG for 5 min before adding the DNA. As positive control htop1 was incubated with 400 μM of CPT. The samples were immediately analysed on 1% agarose gel in TBE buffer, both supplemented with 0.5 μg/ml EtBr.

### Cell viability assay

Different tumour cell lines were seeded in a 96‐well plate for 24 h at 37 °C, 5% CO_2_ to evaluate cell viability as previously described^16^. Cells were treated with different amounts of MG or Hycamtin (topotecan, positive cytotoxicity control), ranging from 12.5 μM to 100 μM. As a control, the cells were treated with the same amount of DMSO. The plates were incubated for 48 h at 37 °C under 5% CO_2_, the medium was then removed and replaced with 200 μL of fresh media supplemented with 0.5 mg/mL of MTT reagent. Samples were incubated again for 4 h in an incubator at 37 °C, 5% CO_2_. Before measuring the absorbance at 570 nm, the medium was replaced with 100 μL of DMSO. Statistical analysis was evaluated by GraphPad Prism using a two-way ANOVA test, and the EC50 value was calculated by nonlinear regression analysis.

## Results

### Membranoid G inhibits the catalytic activity of htop1

The inhibitory effect of MG on htop1 activity was assessed by a plasmid relaxation assay (Figure 1(B–D)). Purified protein was incubated with a supercoiled plasmid in the absence or presence of an increasing concentration of MG for 1 h. Samples were analysed by electrophoresis on agarose gel. The results indicate that MG inhibits the relaxation activity of htop1 in a dose dependent manner and also as a function of the pre-incubation (Figure 1(C,D)). In fact, simultaneous addition of enzyme, MG and DNA determines an inhibition of the relaxation activity from 150 μM MG (Figure 1(B, lane 8)) and is maximal at a drug concentration of 200 μM (Figure 1(B, lane 9)), although a complete inhibition is never achieved under these conditions. The assay, carried out after pre-incubating the enzyme with increasing concentrations of MG before the addition of DNA, shows a greater inhibitory effect on htop1 activity, with a strong inhibition starting from 80 μM (Figure 1(C, lane 6)). As a control, to ensure that MG does not affect the electrophoretic mobility of DNA, the substrate has been incubated in the absence of htop1 and in presence of the compound (Figure 1(B,C, lane 10)). Since MG is dissolved in DMSO, as additional control, enzyme activity was evaluated in the presence of an identical amount of DMSO without MG, to show that DMSO does not affect the relaxation activity of htop1 (Figure 1(B,C, lane 1)).

To further investigate MG behaviour, we carried out two relaxation assays as a function of time to understand whether MG inhibits htop1 catalytic activity in a reversible or irreversible manner, choosing a concentration of 100 μM. The first experiment was performed pre-incubating the enzyme while the other one by simultaneously adding the compound. The result confirms that pre incubation of htop1 with the MG completely inhibited the catalytic activity of enzyme (Figure 1(D, lanes 10–18)) while when MG was simultaneously incubated to the reaction mixture, the inhibition was reduced (Figure 1(D, lanes 19–27)). In both cases we found that MG acted as an irreversible drug, as evidenced by the fact that the inhibition is constant all over time. The time course assay was performed in the presence of DMSO, to confirm that the solvent has no inhibitory effect (Figure 1(D, lanes 1–9)).

### Cleavage assays in the absence and presence of MG

**Figure 2.**
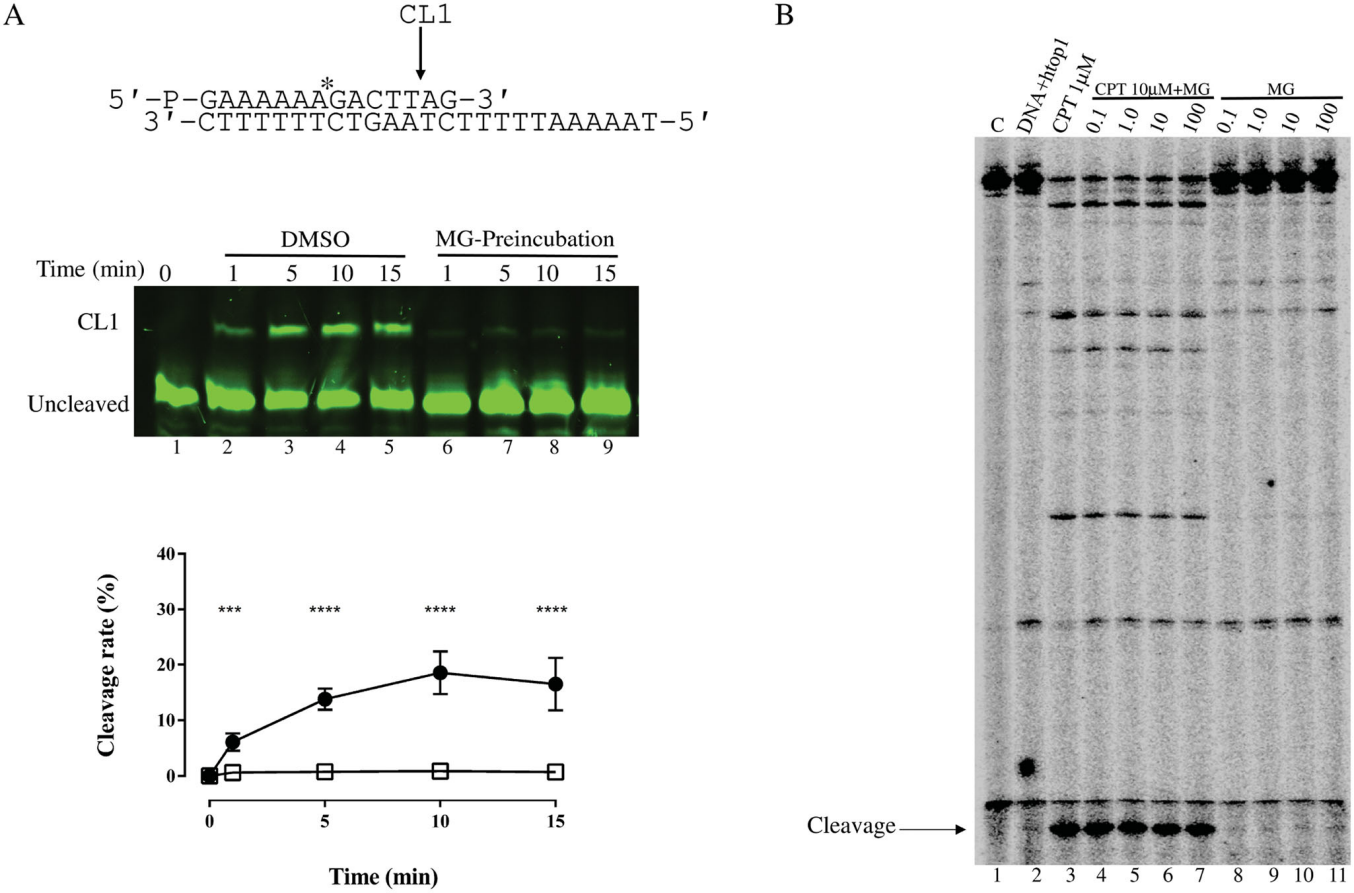
Cleavage and religation kinetics. (A) Top panel: cleavage reaction as a function of time of the CL14/CP25 SS, depicted on the top of the figure, in presence of DMSO (lanes 2–5) and after MG pre-incubation (lanes 6–9). In lane 1 the protein has not been added. CL1 represents the DNA strand cleaved by the enzymes at the preferred cleavage site, indicated by an arrow. Bottom panel: percentage of cleaved SS, plotted against time for the reaction with DMSO (circle) and after MG pre-incubation (triangle). Data shown are means ± SD of 3 independent experiments and were analysed by a two-way ANOVA test. ^****^p < 0.0001 and ***p < 0.001. (B) htop1 cleavage assay gel. From left to right: Lane 1, only DNA, lane 2, htop1 + DNA, lane 3 CPT 1 µM, lanes 4–7, 10 µM CPT + MG (0.1, 1.0, 10, and 100 µM), lanes 8–11, MG (0.1, 1.0, 10, and 100 µM).

To characterise which step of htop1 catalytic mechanism is affect by MG, the activity of enzyme was evaluated on a suicide substrate (SS) in absence and presence of MG in a time course experiment, pre-incubating the drug with the protein. The experiment was done with a fluorescently labelled SS made by the CL14-FITC annealed to the CP25-P oligonucleotide phosphorylated at the 5′ end to produce a duplex with a 5′ single-strand overhang (Figure 2(A)). This substrate allows the enzyme to generate a suicide product since the cleaved AG-3′ dinucleotide is too short to be religated, leaving the enzyme covalently attached to the oligonucleotide 3′-end. 1.2 pmol of enzyme, were incubated with 100 µM MG and the reaction was stopped at different time points from 1 to 15 min, precipitating the samples in 100% ethanol followed by digestion with trypsin. The products resolved on a denaturing urea polyacrylamide gel indicate that the cleavage is inhibited (Figure 2(A lanes 6–9)) while in its absence and in presence of only DMSO (lanes 2–5) the enzyme is cutting as indicated by the plot of the percentage of the cleaved fragment (CL1) against time (Figure 2(A bottom panel)).

To investigate the effect of MG on the cleavage/religation equilibrium, the stability of the covalent DNA–enzyme complex was analysed using a double stranded DNA substrate, radiolabelled on one of the 3′ ends. When the enzyme was incubated with DMSO (Figure 2(B, lane 2)) a very small amount of the cleaved DNA strand was detected at the preferred DNA cleavage site, as expected and indicated by the arrow. When htop1 was exposed to CPT, a dramatic increase of the cleaved DNA fragment was observed (Figure 2(B, lane 3)), indicating that the equilibrium is shifted towards cleavage, as the drug reversibly binds to the covalent DNA–enzyme complex slowing down the religation^29^. When the protein was incubated with increasing concentration of MG, in presence of CPT, the band of the cleaved fragment was still observed indicating that the enzyme was cleaving the substrate permitting the CPT to stabilise the cleaved complex (Figure 2(B, lanes 4–7)). When the protein was incubated with MG alone, the band of the cleavable complex was not observed (Figure 2(B, lanes 8–11)), indicating that the drug is not inhibiting the religation. This experiment opens the possibility of two different scenarios: MG is unable to induce htop1-mediated DNA cleavage or, as suggested by the cleavage assay in Figure 2(A), the compound is inhibiting protein binding.

### DNA mobility shift assay

**Figure 3.**
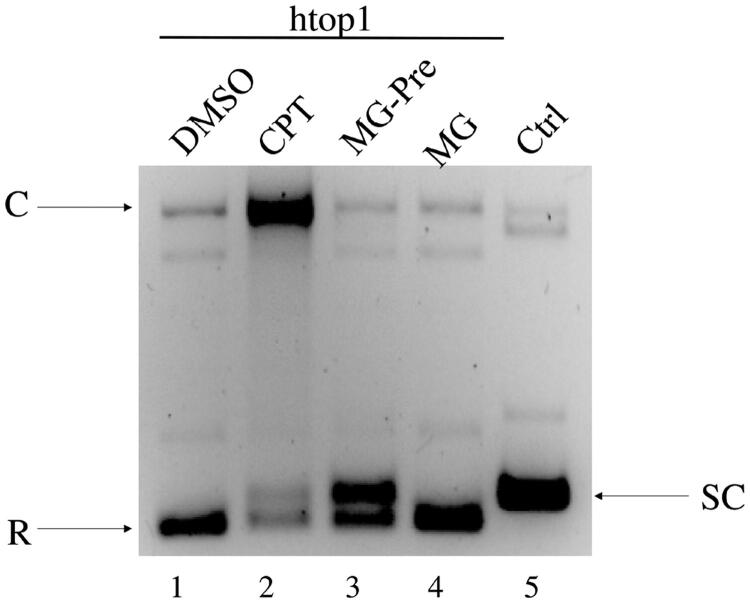
DNA mobility shift assay. Lane 1, pBlueScript DNA and htop1; lane 2, pBlueScript DNA, htop1 and CPT; lane3, pBlueScript DNA, htop1 after MG pre-incubation and lane 4 in simultaneous addition; lane 5, pBlueScript DNA only. SC: supercoiled DNA; R: relaxed DNA; C: cleavable complex htop1-DNA-drug.

The cleavage inhibition displayed by MG with htop1 (Figure 2(A, lanes 6–9 and B, lanes 7–10)) may be due either to a catalytic inhibition of the cleavage reaction or to a prevention of htop1 binding to its DNA substrate. In order to clarify this point, a DNA mobility shift assay was carried out. The results (Figure 3) indicate that when the enzyme is pre-incubated with MG (lane 3) there is a partial inhibition of the binding of htop1 to the substrate, as demonstrated by the presence of both the supercoiled DNA (SC) and the relaxed DNA (R). On the other hand, only the relaxed DNA is observed upon a simultaneous addition of the enzyme, MG and the supercoiled DNA substrate (lane 4) as it is when only htop1 is added to the substrate (lane 1). In the presence of CPT, which inhibits the religation activity of htop1 without interfering with the binding step of htop1 to DNA, a strong band typical of the cleaved complex was observed (lane 2).

### Cell viability assay

**Figure 4.**
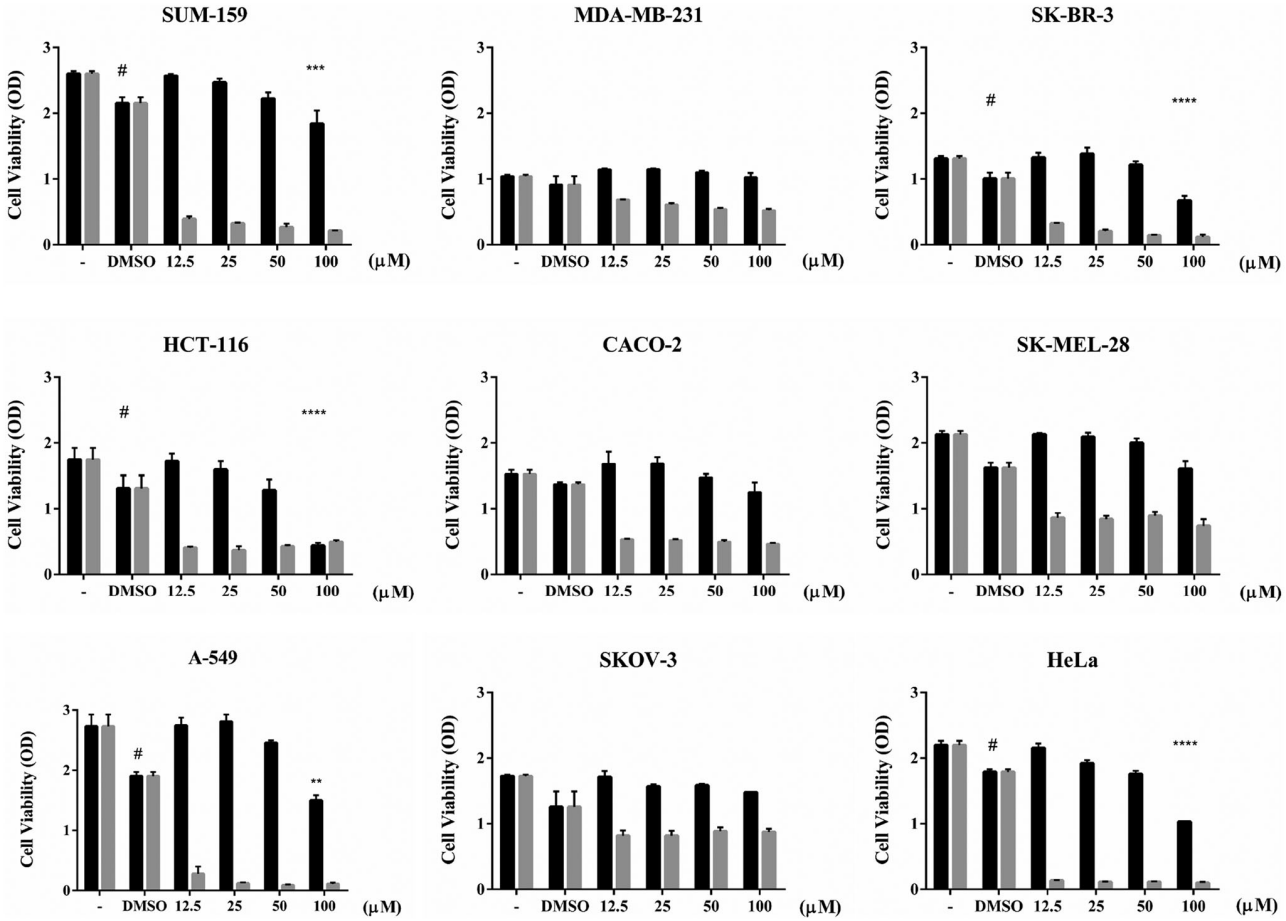
Cell viability assay on cancer cell lines in presence of MG and TPT. Cytotoxicity of MG (black) and TPT (gray) were tested on several cancer cell lines indicated on the top of the histograms using MTT reagent. TPT is the standard positive cytotoxicity control. The reported data represent three independent experiments with mean ± SD values analysed by a two-way ANOVA test. ^#^control for comparison test, **p < 0.01, ***p < 0.001 and ^****^p < 0.0001.

MG showed an inhibitory effect on the htop1 relaxation and cleavage activity, and a cell viability assay was carried out on several cancer cell lines to investigate its potential cytotoxic activity. Cancer cells were treated for 48 h with different concentrations of MG ranging from 12.5 µM to 100 µM (Figure 4). Among the tested cancer cell lines, HeLa, SUM-159, SKBR-3, HCT-116 and A-549 cells show a significant reduction of viability at 100 µM compared to control cells in presence of DMSO alone, while CACO-2, SKOV-3 and MDA-MB-231 were no affected by MG. All the cells treated with TPT, that is selectively targeting htop1 and it is routinely used as positive control, had a strong viability reduction. To better evaluate cytotoxicity of MG, it was calculated the effective concentration of cell growth inhibition (EC50) relative to control with the solvent but without the compound. As shown in Table 1, MG exhibited a low cytotoxic effect for most cancer cell lines, with a EC50 values ranging from 0.007 µM to 1.5 mM.

**Table 1.** The reported data consist of effect concentration values (EC50) for each cell lines at which the concentrations of compound that resulted in 50% cell growth inhibition.

Panel	Cell Line	EC50 (µM)
Breast cancer	SUM-159	80
	MDA-MB-231	52
	SKBR-3	54
Colon cancer	HCT-116	1500
	CACO-2	50
Melanoma	SK-MEL-28	1200
Non-small cell lung cancer	A-549	53
Ovarian cancer	SKOV-3	0.007
Endocervical adenocarcinoma	HeLa	105

## Discussion

Nature has been a source of drugs for the treatment of many human diseases^30^ and most of the drugs now available come from NPs. To find new NPs with novel characteristic we decided to screen, against htopo1, compounds derived from organisms living in Antarctica. Indeed, there are several NPs obtained from Antarctic organism that display interesting therapeutic effect. Among them, Antartina isolated from the Antarctic plant *Deschampsia antarctica* displays antitumor activity^31^, Variolin B, from the Antarctic sponge *Kirckpatrickia variolosa* has antitumor and antiviral properties and a subclass of pyrroloiminoquinone alkaloids extracted from the Antarctic sponge *Latrunculia biformis*, exhibits strong antitumor activity against different cancer types^17^.

In our screening against htopo1 we found interesting inhibitory activity from MG, a diterpene from the Antarctic sponge *Dendrilla antarctica*. This compound has also been reported to have strong effect on *Leishmania donovani* infected macrophages, but its mechanism of action has not been elucidated^25^. Here we show an *in vitro* activity against htop1, an ubiquitous enzyme present also in bacteria, virus and parasites such as *Plasmodium falciparum* and *L. donovani,* causing malaria and human visceral leishmania respectively^32^. Our results show that MG, when pre-incubated with the enzyme inhibits htop1 in an irreversible manner by binding to DNA. The metabolite has also a cytotoxic effect on cancer cell lines whose doubling time is below 30 h, as SUM-159, SK-BR-3, HCT-116 and HeLa cells. A possible hypothesis is that cells having a fast replication rate requires a high htop1 activity^33^, thus explaining the cytotoxicity and the inhibitory activity of MG against htop1. However, the wide range of EC50 values, for all cell lines (Table 1) suggest the presence other targets beside htop1. These high concentrations required to inhibit cell growth could be explained by the fact that the MG affect multiple targets reducing its effect on htop1. The reported data show for the first time, the effect of MG on htop1 and on tumour cells, suggesting its possible use as a lead compound to develop new anticancer drugs.
